# Evaluation of the new advanced 15-loci MIRU-VNTR genotyping tool in *Mycobacterium tuberculosis *molecular epidemiology studies

**DOI:** 10.1186/1471-2180-8-34

**Published:** 2008-02-24

**Authors:** Noelia Alonso-Rodríguez, Miguel Martínez-Lirola, Marta Herránz, Marisa Sanchez-Benitez, Pilar Barroso, INDAL-TB group, Emilio Bouza, Darío García de Viedma

**Affiliations:** 1 Servicio de Microbiología y Enfermedades Infecciosas. Hospital Gregorio Marañón, Universidad Complutense, Madrid, CIBER de Enfermedades Respiratorias (CIBERES), Spain; 2 Complejo Hospitalario Torrecárdenas, Almería, Spain; 3 Unidad de Tuberculosis de Poniente, Almería, Spain; 4 Delegación de Salud Distrito Levante, Almería, Spain

## Abstract

**Background:**

During the last few years, PCR-based methods have been developed to simplify and reduce the time required for genotyping *Mycobacterium tuberculosis *(MTB) by standard approaches based on *IS6110*-Restriction Fragment Length Polymorphism (RFLP). Of these, MIRU-12-VNTR (Mycobacterial interspersed repetitive units- variable number of tandem repeats) (MIRU-12) has been considered a good alternative. Nevertheless, some limitations and discrepancies with RFLP, which are minimized if the technique is complemented with spoligotyping, have been found. Recently, a new version of MIRU-VNTR targeting 15 loci (MIRU-15) has been proposed to improve the MIRU-12 format.

**Results:**

We evaluated the new MIRU-15 tool in two different samples. First, we analyzed the same convenience sample that had been used to evaluate MIRU-12 in a previous study, and the new 15-loci version offered higher discriminatory power (Hunter-Gaston discriminatory index [HGDI]: 0.995 *vs *0.978; 34.4% of clustered cases *vs *57.5%) and better correlation (full or high correlation with RFLP for 82% of the clusters *vs *47%). Second, we evaluated MIRU-15 on a population-based sample and, once again, good correlation with the RFLP clustering data was observed (for 83% of the RFLP clusters). To understand the meaning of the discrepancies still found between MIRU-15 and RFLP, we analyzed the epidemiological data for the clustered patients. In most cases, splitting of RFLP-clustered patients by MIRU-15 occurred for those without epidemiological links, and RFLP-clustered patients with epidemiological links were also clustered by MIRU-15, suggesting a good epidemiological background for clustering defined by MIRU-15.

**Conclusion:**

The data obtained by MIRU-15 suggest that the new design is very efficient at assigning clusters confirmed by epidemiological data. If we add this to the speed with which it provides results, MIRU-15 could be considered a suitable tool for real-time genotyping.

## Background

Genotyping methods have been extensively applied to analyze the recent transmission dynamics of *Mycobacterium tuberculosis *(MTB). *IS6110*-restriction fragment length polymorphism (RFLP) is the reference technique for genotyping MTB [1] because of its high discriminatory power. However, the need for well-grown cultures and purified DNA to obtain RFLP data means that it takes a long time to produce results. In addition, analysis of RFLP band-patterns requires specific software, which makes it difficult to interpret and exchange data. Finally, RFLP is limited when analyzing MTB strains with a low number of *IS6110 *copies.

Different PCR-based genotyping approaches targeting the variable number of tandem repeats (VNTR) have been developed to compensate for the limitations of RFLP. These include VNTR analysis based on mycobacterial interspersed repetitive units (MIRU) [2,3], which has been considered a good alternative to the reference method and has proven to be faster and easier to perform. MIRU-VNTR genotyping based on a 12-loci set (MIRU-12) has been evaluated in several studies in different settings. Some authors [2,3] have found it to show a discriminatory power equivalent to that of RFLP and have considered it an alternative to *IS6110*-RFLP for epidemiological purposes [3-6]. However, other authors have found limitations in its discriminatory power and incomplete correlation with the RFLP analysis [7-9], indicating that MIRU analysis should be combined with an additional genotyping method [9-12].

Recently, a new MIRU-VNTR format has been developed to improve the discriminatory power of MIRU-12 [13]. This new version targets 15 loci (6 from the previous 12-loci version and 9 new ones), although to date few studies have evaluated its efficiency [13,14]. We compared the new 15-loci format of MIRU-VNTR (MIRU-15) with the MIRU-12 version and IS6110-RFLP in two independent samples in the context of molecular epidemiology studies. These data could serve to clarify the final application of this new procedure in our setting.

## Results

### I) MIRU-15 analysis of a convenience sample

#### a) Clustering rates and discriminatory power

**Figure 1:**
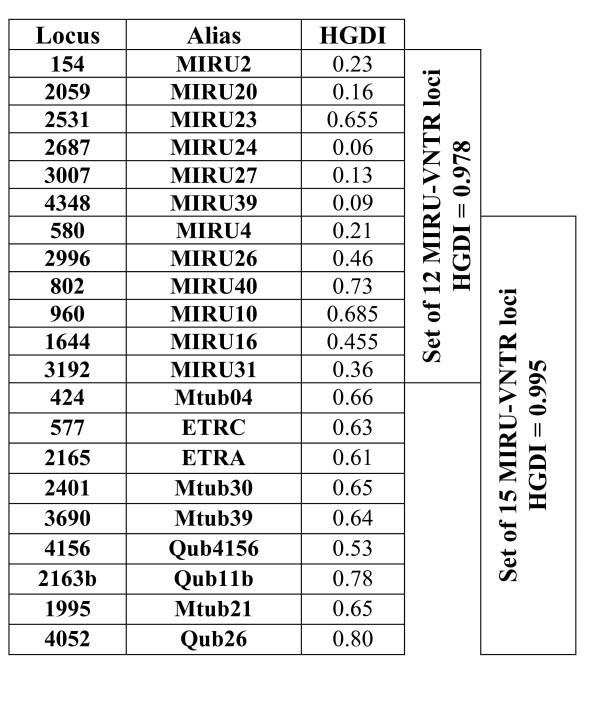
**Hunter-Gaston discriminatory index (HGDI) for the loci in MIRU-12 and MIRU-15 sets**. The HGDI of each locus was calculated based on the convenience sample of 134 isolates.

As an initial approach to comparing the efficiency of MIRU-15 with MIRU-12, we studied the same convenience sample we had already used to test the efficiency of MIRU-12 [7]. Spoligotyping indicated that most of the isolates corresponded to the LAM (32.1%) and Haarlem (28.4%) lineages. Only 2.2% of the isolates belonged to the Beijing family. RFLP clustered 41% of the isolates in 17 clusters (2 to 9 representatives), whereas MIRU-12 clustered 57.5% in 20 clusters (2 to 14 representatives). MIRU-15 clustered 34.4% of the isolates in 16 clusters (2 to 5 representatives). Therefore, the Hunter-Gaston discriminatory index (HGDI) was higher for MIRU-15 (0.995) than for MIRU-12 (0.978). The loci with the highest HGDIs were QUB26 (0.8), QUB11b (0.78), and MIRU40 (0.73), two of which were not present in the MIRU-12 version (Figure 1). If we consider the nine new loci that were not included in the MIRU-12 version, all of them showed HGDI values over 0.5, whereas in MIRU-12 only three (MIRU10, MIRU16, and MIRU40) showed equivalent values and the remainder showed HGDI values below 0.25 (Figure 1).

#### b) Correlation analysis

**Figure 2:**
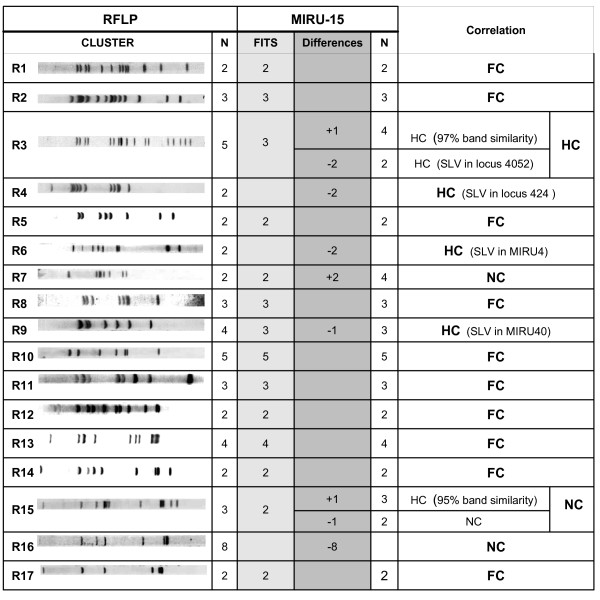
**Comparative analysis between RFLP and MIRU-15 in the convenience sample**. N indicates the number of isolates clustered by RFLP or MIRU. The FITS column indicates the number of isolates grouped both by RFLP and MIRU-15. The Differences column indicates the number of isolates clustered by MIRU and unclustered by RFLP (+N) or the number of isolates clustered by RFLP and unclustered by MIRU (-N). The Correlation column specifies whether correlation is Full Correlation (FC), High Correlation (HC) or No Correlation (NC). For the isolates clustered with high correlation, the single locus variation (SLV) or percentage of *IS6110 *band similarity is specified.

The correlation between RFLP and MIRU-15 was higher than that observed with MIRU-12 in our previous study [7]. MIRU-15 showed full or high correlation in 14/17 (full correlation in ten and high in four) of the clusters defined by RFLP, whereas MIRU-12 had shown an equivalent correlation in only 8/17 clusters (seven with full correlation and one with high correlation) (Figure 2).

**Figure 3:**
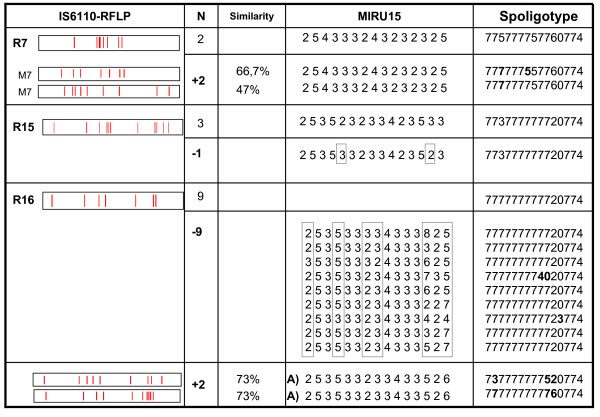
**Detailed analysis of the discrepant cases in the No Correlation (NC) clusters**. RFLP clusters are defined as Rn. M7: isolates grouped by MIRU together with the two isolates grouped in cluster R7. "A" indicates the cluster defined by MIRU but not by RFLP. N indicates the number of clustered isolates: The number of isolates clustered by MIRU and unclustered by RFLP (+N), or the number of isolates clustered by RFLP and unclustered by MIRU (-N) are highlighted in bold. MIRU loci showing differences are boxed. Spoligotypes are shown using octal code; differences are highlighted in bold.

MIRU-15 showed noncorrelation with the RFLP data in only three clusters (Figure 3), whereas MIRU-12 had shown no correlation much more frequently. The discrepancies shown by MIRU-15 corresponded to i) the addition of two isolates to an RFLP cluster (cluster R7), although with low *IS6110 *band similarities (66.7% and 47%), ii) the discrimination of an isolate with a double locus variation (DLV) (cluster R15), and iii) full splitting (differences in more than two loci) of the largest cluster defined by RFLP (cluster R16 which included nine isolates). Additionally, in one of these clusters with no correlation (cluster R15), MIRU-15 grouped with high correlation another isolate that showed 95% *IS6110 *band similarity (Figure 2).

MIRU-15 also defined a new cluster (cluster A) between two isolates with low *IS6110 *band similarity (73%) (Figure 3). In our previous study [7], MIRU-12 defined up to eight clusters not assessed by RFLP.

When spoligotyping was applied as a second-line method in discrepant cases, all the isolates grouped by MIRU-15 that were unclustered by *IS6110*-RFLP (cluster R7 and cluster A) were split. Cluster R16, which was subdivided by MIRU-15, was split by spoligotyping (Figure 3).

### II) MIRU-15 analysis of a population-based sample

After observing that MIRU-15 improved the MIRU-12 data, we decided to re-evaluate MIRU-15 efficiency with an independent sample. Unlike the first convenience sample, the new one included all the MTB isolates in Almería during the period 2003–2006. In total, 308 isolates were genotyped, corresponding to 140 autochthonous cases (45.4%) and 168 immigrant cases (546%). The most widely represented countries of origin in the study population were Morocco (71 cases, 42.2%), Romania (22 cases, 13%), and Mali (13 cases, 7.7%).

#### Correlation analysis

**Figure 4:**
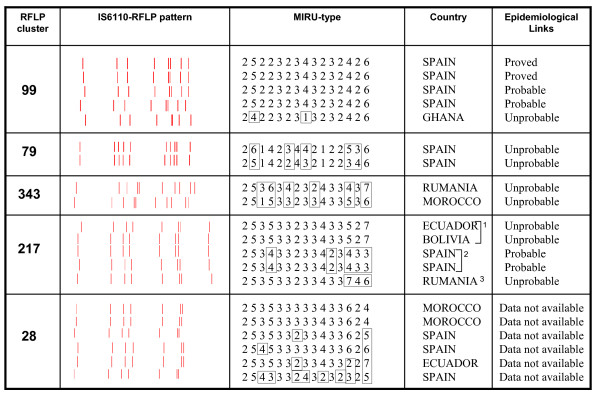
**Discrepancies between RFLP clustered cases and MIRU-15 data in the population sample**. MIRU loci showing differences are boxed. The last column shows the epidemiological evaluation of the clustered cases. Cluster 217 was subdivided by MIRU-15 into three different MIRU types (marked with numbers 1, 2, and 3).

When the RFLP-clustered cases from Almería were analyzed by MIRU-15, full or high correlation was obtained in 83% of the clusters (24/29 clusters; 20 full and four high). MIRU-15 showed no correlation in the remaining five RFLP clusters (Figure 4), and discriminated 12/20 isolates grouped in them (13.2% of the total of the isolates genotyped in the study). The discrepancies were as follows: i) one isolate clustered by RFLP (cluster 99) showed a DLV (one repetition in MIRU26 and three repetitions in locus 577); ii) splitting of two RFLP clusters (cluster 79 and cluster 343) with isolates showing differences in more than four loci: and iii) three and four isolates clustered by RFLP (in clusters 217 and 28, respectively) showed differences in two to eight MIRU-15 loci. Additionally, MIRU-15 subdivided RFLP cluster 217 into three different MIRU types.

In order to understand the significance of the concordances and discrepancies between the clusters defined by RFLP and MIRU-15, we evaluated the epidemiological evidence found for these clusters. Therefore, we labeled the clusters according to a gradient of evidence of epidemiological links between their cases (proved, probable, or improbable). For the RFLP clusters with full or high correlation with MIRU data, we obtained epidemiological data in 22/24 clusters. Of these, epidemiological evidence (values 1 or 2) was detected in 16/22 clusters (72.7%). For the 71 patients included in these 22 RFLP clusters, we detected epidemiological links for 43/59 cases with available epidemiological data (84.3%, 31 with value 1 and 12 with value 2). For the remaining 15 cases no epidemiological evidence was found.

Epidemiological data were available for four of the five clusters that were split by MIRU-15, and no links were found for all the representatives which were discriminated by MIRU-15 (Figure 4). A review of these cases reveals the following: a) In cluster 99 (eight-band pattern), the only isolate without epidemiological links was clearly split by MIRU-15 (differing in two loci). This corresponded to an unrelated Ghanian patient in a cluster of Spanish cases; b) In a further two clusters (high-copy banded) involving two cases each (clusters 79 and 343), MIRU-15 showed discrepancies in five and six loci respectively, and once again, no epidemiological connections were found; c) No epidemiological links could be found for the remaining cases involved in cluster 28 (differing in two to eight loci); d) Cluster 217 (eight-band pattern), involving different nationalities, was also split by MIRU-15, according to the geographic origin/socio-cultural background of the cases (one MIRU profile was shared by an Ecuadorian and a Colombian patient, the second one grouped two Spanish patients, and the third belonged to a Romanian patient). No links were found between the multinational patients grouped in RFLP cluster 217, but links were found between the cases in at least one of the split subgroups (the Spanish cluster).

## Discussion

During the last few years, the search for an alternative to RFLP, has led to the development of a PCR-based technique, MIRU-VNTR [2,3,5]. This technique has proven to be fast and easy to perform, and it allows the direct exchange of data between laboratories. Different combinations of MIRU and other VNTR loci have been published [9-11,15,16], but most of the studies have focused on a set of 12 MIRU loci [6,8,12,17-20]. This format offers a higher discriminatory power (close to the gold standard *IS6110 *RFLP) than other PCR-based genotyping techniques [4-6,16,20]. Other studies [7,8,12,21], however, have revealed lower discriminatory power and a low correlation with RFLP data, which improved if complemented with spoligotyping. In a previous study [7], we focused on isolates with high-copy-number fingerprints, to try to evaluate MIRU-12 in a more challenging situation than that of studies performed in circumstances that could favor MIRU, e.g. high proportion of low-copy-number RFLP fingerprints, low variability of circulating strains due to the prevalence of specific genetic families, etc. We found that, compared with RFLP, MIRU-12 overestimated candidates for recent transmission, by grouping a higher number of isolates and defining a higher number of clusters. Furthermore, 53% of MIRU-12 clusters showed a low or no correlation with RFLP data. These data urge caution when considering substituting RFLP with MIRU-12, and suggest that incongruent study conclusions could be due to geographic differences in the genotypic composition of circulating strains.

Supply et al [13] have recently published a proposal for a new refined set of 24 MIRU-VNTR loci. When the advanced set was tested on 824 isolates including representatives of the main MTB lineages, a subset of 15 loci (MIRU-15) was considered to have the highest efficiency because it contained 96% of the resolution obtained with the whole 24-loci set. However, the MIRU-15 design has received little attention [13,14]; therefore, additional studies in a variety of socio-epidemiological backgrounds are necessary to fully evaluate the usefulness of this new strategy. This was the aim of our analysis. We began by evaluating the new 15-loci MIRU version with the same convenience sample that was used to evaluate MIRU-12. The genetic lineages which were over-represented in this sample were LAM and Haarlem, and only three Beijing strains were found, thus ruling out enrichment in strains which have been found to be poorly discriminated by MIRU-12 [18]. MIRU-15 showed higher discriminatory efficiency by grouping 23.1% fewer isolates than MIRU-12 and showing a higher HGDI value (0.995 vs 0.978 for MIRU-12). This increase in resolution was due to the nine new loci whose HGDI values were higher than 0.5, whereas only three in the previous set achieved equivalent values, which justified the selection of these loci in the new advanced design. With regard to the correlation with RFLP, our previous study revealed that only 8 out of 17 RFLP clusters fitted well with MIRU-12 data, and it was necessary to include spoligotyping to improve these results. MIRU-15 alone increased the number of clusters with good correlation with RFLP data to as many as 14/17.

In this study, we considered full or high values as indicative of a good correlation between MIRU-15 and RFLP. Correlation was considered to be high when MIRU-grouped isolates sharing RFLP patterns showed high *IS6110 *band similarity (≥ 95%), or isolates sharing RFLP types showed subtle differences (single locus variations [SLVs]) in the MIRU analysis. This decision was supported by studies that found epidemiological links for clusters including isolates with subtle differences in RFLP isolates [22-25] and by reports that some MIRU loci have faster molecular clocks [3,9,10,13,19], which could even lead to SLVs between isolates linked to an ongoing transmission event.

Although good correlation was generally found in our convenience sample, no correlation with RFLP was observed for three RFLP clusters. MIRU-15 added two isolates in the R7 RFLP cluster, which showed only low *IS6110* band similarity percentages with the other clustered isolates. The spoligotypes for these two isolates were also different from the R7 representative pattern, although they belonged to the same genetic lineage (T5 family). This could be due to the fact that MIRU-15 misassigned these isolates and we were probably observing a genotypic convergence phenomenon. On the other hand, the rate of MIRU changes is relatively lower than that of *IS6110*-RFLP and spoligotyping, so it could also be possible that the MIRU type in this case is shared by different representatives of the T5 lineage. In the remaining two noncorrelated clusters, we observed that MIRU-15 split them to a different extent, and we detected differences (in more than two loci) among isolates sharing an RFLP type. In addition, the largest RFLP cluster was fully split by MIRU-15 (spoligotyping also split this cluster) with differences in 2–5 loci for all the isolates. Splitting of RFLP clusters by MIRU-15 has been found in clusters involving strains with low-copy-band fingerprints (< 6 bands) [6,15,17,26,27], which is somehow expected, although in this case the strain had seven bands. Splitting of RFLP clusters, even those with high-copy-band strains has been described elsewhere [13], and these findings urge caution in assuming certainty for all defined clusters.

In order to fully understand the meaning of the concordances and discordances between RFLP and MIRU-15 data, we used an unselected population-based sample with available epidemiological data to interpret potential discrepancies. We chose the MTB isolates cultured in the province of Almería during a 2.5-year period to ensure quality in the clustering assignation and to increase the observation time of other studies [14]. We decided not to consider the orphan cases (unclustered by RFLP) and to focus exclusively on the analysis of the RFLP clusters detected in this population sample because they have epidemiological value, as they are used as markers for recent transmission. The ability of MIRU-15 to classify as orphan those cases unclustered by RFLP is being evaluated in an ongoing prospective population-based study.

As with our findings in the convenience sample, the correlation between MIRU-15 and RFLP genotypes was good; full or high correlation with the RFLP data was detected for 24 of the 29 clusters genotyped by MIRU-15. We found epidemiological links in 72.7% of the clusters and in 84.3% of the cases with available epidemiological data. These percentages of "epidemiologically proved" clusters agree with those of other studies, and it is well known that higher values are only obtained if highly refined epidemiological surveys are followed up [28-30]. It is noteworthy that almost all the clusters (5/6) without epidemiological links involved two cases, and it is generally assumed that the detection of links in transmission chains involving a reduced number of cases produces a lower yield. It is interesting that, of the four RFLP clusters with epidemiological information available which were split by MIRU-15, no links were found for all the representatives which were discriminated by MIRU-15. This suggests that MIRU-15 was able to detect some cases that were falsely clustered by RFLP. Moreover, in at least one case, MIRU-15 redefined epidemiologically-consistent subclusters within a common RFLP cluster which was not epidemiologically supported.

## Conclusion

The data obtained by MIRU-15 in this study and elsewhere [14] suggest that the new design is very efficient at assigning clusters confirmed by epidemiological data. If we add this to the speed with which it provides results, MIRU-15 could be considered a suitable tool for real-time genotyping. This could be essential in study populations such as ours, which is undergoing an epidemiological transformation due to the marked increase in tuberculosis among immigrants. The complexity of this situation reduces the efficiency of standard epidemiological approaches and demands new strategies such as MIRU-15 to allow rapid identification of clusters.

## Methods

### Sample

Clinical specimens were processed according to standard methods and grown in Lowenstein-Jensen slants and in MGIT (Becton Dickinson, Sparks, Maryland, USA) liquid media.

#### Convenience sample

This was composed of 134 MTB isolates from independent patients in three institutions in Almería (southeast Spain). The isolates had been previously analyzed using a set of 12 MIRU-VNTR loci and *IS6110*-RFLP [7].

#### Population-based sample

From January 2003 to June 2006, 353 MTB isolates were cultured from independent patients (60% of all diagnosed TB cases) from the three public hospitals in the province of Almería (635,850 inhabitants) and 308 of these (87.2%) were genotyped by RFLP. From this sample, we selected the 91 isolates clustered by RFLP. All the isolates analyzed had patterns with more than six *IS6110 *copies.

### Molecular typing methods

DNA extraction, *IS6110*-RFLP typing, and spoligotyping were performed according to standard methods [1]. MIRU-VNTR was performed by amplifying the 15 MIRU-VNTR loci as described elsewhere [13] with some modifications: DMSO was added to the PCR mixtures instead of Q-solution (4% DMSO for MIRU loci 580, 2996, 802, 960, 1644, and 3192, and 12% DMSO for the remaining loci). MIRU loci 580, 2996, 802, 960, 1644, and 3192 were amplified using two multiplex-PCRs and fragment sizes were analyzed by GeneScan™ 2500 ROX™ Size Standard and the ABIPRISM 3100 genetic analyzer (Applied Biosystems, Foster City, California, USA). The remaining loci were amplified individually and PCR products were separated by electrophoresis at 45 V for 17 h 30 min, using MS8 2% agarose gels (Pronadisa, Madrid, Spain). Fragment sizes were calculated with the ChemiDoc system (BioRad, Hercules, California, USA) and the Diversity database (BioRad), using a 100-bp ladder (Invitrogen, Carlsbad, California, USA) as a molecular weight marker. The number of repeats in each locus was calculated by applying the corresponding conversion tables (P. Supply, personal communication).

The MIRU-type was defined after combining the results for the 15 loci in the following order: 580 (MIRU4), 2996 (MIRU26), 802 (MIRU40), 960 (MIRU10), 1644 (MIRU16), 3192 (MIRU31), 424 (Mtub04), 577 (ETRC), 2165 (ETRA), 2401 (Mtub30), 3690 (Mtub39), 4156 (QUB4156), 2163b (QUB11b), 1995 (Mtub21), and 4052 (QUB26).

### Molecular typing analysis

#### Cluster definition

Genotypes were analyzed using Bionumerics 4.6 (Applied Maths, Sint-Martens Latem, Belgium). Clusters were defined when 100% *IS6110 *band similarity was observed between patterns. *IS6110*-RFLP, spoligotyping, and MIRU-VNTR dendrograms were generated using the unweighted pair group method with arithmetic averages (UPGMA). The Dice coefficient was used with RFLP and the categorical coefficient with spoligotyping and MIRU.

#### Correlation analysis between clusters obtained by different techniques

Correlation between MIRU-VNTR and *IS6110*-RFLP analysis was defined as follows:

a) Full correlation, when the isolates clustered by *IS6110 *RFLP and MIRU shared identical patterns.

b) High correlation, when we detected an SLV by MIRU (one or two alleles of difference in a single locus) in the cluster defined by RFLP or some MIRU-clustered isolates not sharing identical RFLP genotypes but with a high *IS6110 *band similarity (≥ 95%).

c) No correlation, when RFLP-clustered isolates were clearly split by MIRU (differences in more than one locus) or MIRU-clustered isolates were clearly different by RFLP (similarities < 95%).

#### Discriminatory power

The Hunter-Gaston discriminatory index (HGDI) was calculated as described elsewhere [31].

### Epidemiological analysis of clusters

In order to evaluate the epidemiological support of clusters in the population sample in Almería, we retrospectively analyzed data from the clinical charts and from standardized interviews with the patients. From these data we defined a scale of epidemiological evidence for the clustered cases based on Supply et al [13] as follows:

Value 1: proved, when the standard epidemiological survey or the interview of the clustered cases revealed the existence of proven links between the cases.

Value 2: probable, when the standard epidemiological survey of the clustered cases revealed the existence of likely links between the cases.

Value 0: improbable, when the standard epidemiological survey of the clustered cases revealed lack of links between the cases.

Value -1: epidemiological data unavailable.

## Authors' contributions

NAR designed the study, performed all the MIRU-VNTR experimental assays, and was involved in the analysis of RFLP, spoligotyping, and MIRU-VNTR results. She analyzed the results and produced the first version of the manuscript. MML analyzed all the clinical and epidemiological data of the Almería sample and he was involved in the analysis of the correlations between RFLP and MIRU in the population sample. MH was involved in the analysis of the isolates by RFLP and spoligotyping. MSB and PB compiled and analyzed all the clinical and epidemiological data. EB critically reviewed the final version of the manuscript. DGV designed the study, supervised all the experimental work, analyzed the results, corrected and produced the final version of the manuscript.

All authors read and approved the final manuscript.
